# *CDDiscovery*

**DOI:** 10.1038/cddiscovery.2015.2

**Published:** 2015-07-27

**Authors:** J Silke, G Melino

**Affiliations:** 1 The Walter and Eliza Hall Institute, Parkville, Victoria, Australia; 2 Department of Medical Biology, University of Melbourne , Melbourne, Victoria, Australia; 3 Department of Surgery & Experimental Medicine, University Rome Tor Vergata, Rome, Italy; 4 MRC Toxicology Unit, Leicester, UK

Here it is! *CDD* number three! On the shoulders of the success of *Cell Death & Differentiation* (*CDD*), and *Cell Death & Disease* (*CDDis*), together with Nature Publishing Group, we are now launching the third journal of the group, *Cell Death Discovery* (*CDDiscovery*), a dynamic, innovative and creative journal, committed to publishing high-quality, peer-reviewed research and review material.

*Cell Death Discovery* is an international, online-only, open-access journal dedicated to publication of scientific findings at the intersection of biomedical research and cell death with the principal focus of scientifically sound work. At a time when concern about the reproducibility of scientific data has become a significant issue, *Cell Death Discovery* will put procedures in place to help guarantee this fundamental aspect and support solid scientific research. It is committed to the rapid publication of high-quality original papers that relate to these subjects, together with topical, usually solicited reviews, editorial correspondence and commentaries on controversial and scientifically informative issues. Unrestricted access to research findings in *Cell Death Discovery* will foster a dynamic and highly productive dialogue between scientists and clinicians, as well as researchers in industry with a focus on cancer, neurobiology and inflammation research.

Together *Cell Death & Differentiation*, *Cell Death & Disease* and *Cell Death Discovery* constitute the *CDD* brand, hereinafter *CDD*-erc. The *CDD* brand has had strong involvement in scientific communication for many years, having launched three distinct journals: *CDD* in 1994, currently with an impact factor of 8.5, *CDDis*, in 2010, currently with an impact factor of 5.5 and now *CDDiscovery*, launching in 2015. In addition, *CDD*-erc organises and is a proud supporter of many scientific conferences.

In parallel to the *CDD*-erc branding, a guarantee *per se*, *CDDiscovery* is also published by Nature Publishing Group (NPG). NPG uses pioneering technologies, innovative formats and first-class editing to provide cutting-edge, timely information for researchers in the public and private sectors, government agencies, educators, clinicians and the general public. By working with scientists, listening to what they say, and always placing emphasis on quality, NPG is the leading publisher for innovative solutions to scientists' information needs. This new journal provides plenty of scope to trial new publishing ideas, and we have a number of new ideas to try … watch this space! Similar to NPG, our editors, who are all actively publishing scientists themselves, will be keen to engage with and find new ways to serve you, our readers.

As for any new journal, full indexing in PubMed and PubMed Central takes time. *Cell Death Discovery* articles will not be indexed in PubMed right away; however, pending review and acceptance, all past published articles will be indexed retrospectively. For PubMed, according to the NLM website, the journal will need to first publish several months worth of copy, and then go through the normal process of being reviewed, which typically takes about 4–6 months (including decision, notification to the journal, and receipt of files). For PubMed Central, full indexing entails publication of 50 articles, and also a separate data evaluation review, which can take up to 6 months. However, authors with NIH/HHMI funding are able and encouraged to self-archive; this includes uploading manuscripts to PubMed Central before the journal is officially accepted. One particular advantage of being part of the *CDD* family is that we are able to increase the visibility of the articles published in *Cell Death Discovery* using the *CDD* and *CDDis* sister platforms, to compensate for the initial reduced Medline visibility.

Like any respected journal, *Cell Death Discovery* has selected a prestigious group of Associate Editors, appointed from a pool of what their colleagues describe as ‘rising stars’. Importantly, we intend that these editors: R Aqeilan (Jerusalem), M-L Asselin-Labat (Melbourne), N Barlev (Saint Petersburg), Q Chen (Bejing), I Harris (Boston), R Killick (London), C Parish (Melbourne), A Rufini (Leicester), AE Sayan (Southampton), K Schroder (Brisbane), K Simon (Oxford) and M Tavassoli (London) reflect the type of scientist that will submit to *Cell Death Discovery* . They come from around the globe, have a broad range of scientific expertise that is not restricted to cell death, and are dynamic and enthusiastic. They will be a driving engine in our goal of guaranteeing quality and timely decisions (Figure 1).

Why publish in *Cell Death Discovery*? Our goal in setting up this new journal has been to address some of the deficiencies that affect us and our colleagues in the current publishing environment. Some are already being addressed by other journals and we endorse their efforts while feeling confident that we have the ability to directly contribute our own solutions.

## Unbiased Reviews

The problem of biased reviews will be addressed with double-blind peer review, whereby both the author and reviewer identities remain anonymous. By removing any chance of bias the review process will be more effective and efficient.

## Fast turnaround

Lengthy review processes are a real drag on the scientific process and we know of many cases where it has taken over a year from submission to acceptance because of a lack of editorial engagement. *Cell Death Discovery* submissions will, however, be dealt with by strong editorial oversight, and editorial decisions will not be handballed to reviewers. Papers will undergo the usual and well-established peer review process, but the focus will be on technical quality and reproducibility of content.

## Reproducibility

Lack of reproducibility in scientific reporting is an emerging issue that strikes at the essence of science itself. This seems to be driven by the fact that many journals appear to value novelty and significance above solid research. It is also linked to the problem that journals are, in our experience, reticent to publish studies that fail to repeat earlier work. Therefore, we explicitly encourage authors to send us work that either upholds, or fails to uphold, earlier work. Our criteria will be scientific quality, and we will appropriately recognise and discuss both successes and failures.

## Open access

Your articles will be freely available under a creative commons licence allowing global dissemination and distribution via NPG’s publishing platform nature.com.

## Prevention of Publication Ethical issues

Your data will be automatically checked for plagiarism and for image defects by dedicated staff and software, and we will insist on attribution of authorship for each data panel. Combined with a detailed submission checklist, we will take a lead in correctly representing data and attributing responsibility for that data. With these measures we will reduce unwanted and inadvertent errors and weed out malfeasance.

Welcome to our new experiment! As good scientists, we know we should not prejudge the outcome, but just this once we will. We are convinced that with the established *CDD* brand, our Editorial Board with their ideas and willingness to try new approaches and the backing of Nature Publishing Group, *Cell Death Discovery* will rapidly become a huge success.

## Figures and Tables

**Figure 1:**
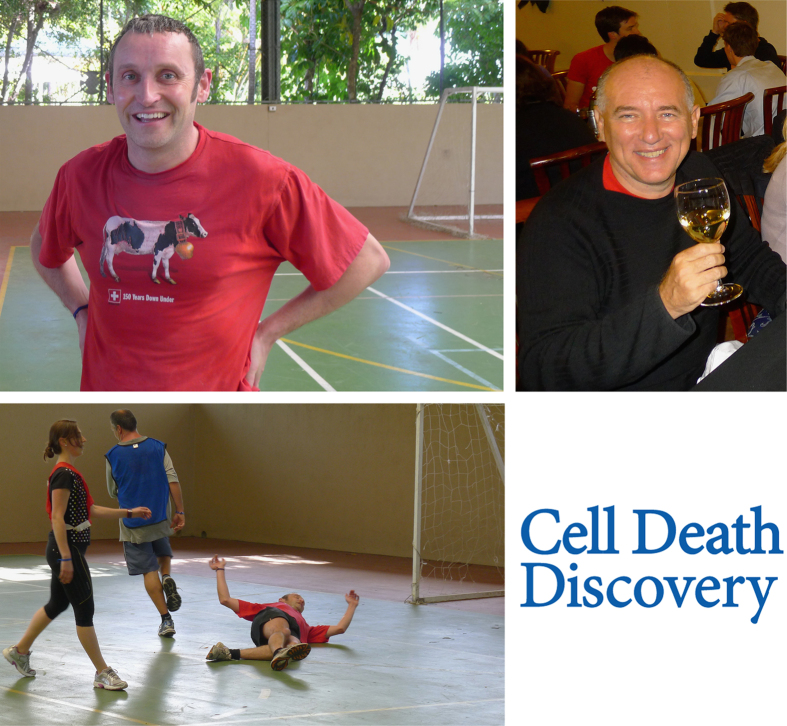
***Cell Death Discovery***, a new challenge, under the lucid guidance of Gerry and the solid standing of John.

